# Biosynthesis and Characterization of Iron Oxide Nanoparticles Using *Chenopodium quinoa* Extract

**DOI:** 10.3390/nano14191607

**Published:** 2024-10-05

**Authors:** Mercedes del Pilar Marcos-Carrillo, Noemi-Raquel Checca-Huaman, Edson C. Passamani, Juan A. Ramos-Guivar

**Affiliations:** 1 Grupo de Investigación de Nanotecnología Aplicada para Biorremediación Ambiental, Energía, Biomedicina y Agricultura (NANOTECH), Facultad de Ciencias Físicas, Universidad Nacional Mayor de San Marcos, Av. Venezuela Cdra 34 S/N, Ciudad Universitaria, Lima 15081, Peru; mercedesdelpilarmc@gmail.com; 2 Centro Brasileiro de Pesquisas Físicas (CBPF), R. Xavier Sigaud, 150, Urca, Rio de Janeiro 22290-180, Brazil; nomifsc@cbpf.br; 3 Departamento de Física, Universidade Federal do Espírito Santo, Av. Fernando Ferrari, 514, Bairro Goiabeira, Vitoria 29075-910, Brazil; edson.caetano@ufes.br

**Keywords:** surface functionalization, quinoa extract, magnetic nanoparticles, biosynthesis, flavonoids

## Abstract

In this study, we achieved the biosynthesis of novel 7–8 nm iron-oxide nanoparticles in the presence of different concentrations (5 to 50% *w*/*v*) of commercial white quinoa extract. Initially, quinoa extract was prepared at various concentrations by a purification route. The biosynthesis optimization was systematically monitored by X-ray diffraction, and the Rietveld quantitative analysis showed the presence of goethite (5 to 10 wt.%) and maghemite phases. The first phase disappeared upon increasing the organic loading (40 and 50% *w*/*v*). The organic loading was corroborated by thermogravimetric measurements, and it increased with quinoa extract concentration. Its use reduces the amount of precipitation agent at high quinoa extract concentrations with the formation of magnetic nanoparticles with hard ferrimagnetic character (42 and 11 emu g^−1^). The enrichment of hydroxyl groups and the negative zeta potential above pH = 7 were corroborated by a reduction in the point of zero charge in all the samples. For alkaline values, the zeta potential values were above the stability range, indicating highly stable chemical species. The evidence of hydroxyl and amide functionalization was qualitatively observed using infrared analysis, which showed that the carboxyl (quercetin/kaempferol), amide I, and amide III chemical groups are retained after biosynthesis. The resultant biosynthesized samples can find applications in environmental remediation due to the affinity of the chemical agents present on the particle surfaces and easy-to-handle them magnetically.

## 1. Introduction

Iron-oxide nanoparticles (IONPs) are extremely small magnetic materials that stand out because of their versatility, as they possess several unique properties. These physicochemical properties include remarkable catalytic capacity, a large specific surface area, and the ability to respond to magnetic fields using permanent magnets [1,2]. Much effort has been put to improve the methods to efficiently produce them, and a promising strategy has been performed to incorporate biological compounds, also known as biological substrates, into their synthesis processes [1,3,4], i.e., a production method known as biosynthesis [1]. For example, Miri et al. performed the biosynthesis of IONPs using a solution derived from the plant *Taranjabin manna* or *Hedysarum* [3]. They found, for example, that these nanoparticles (NPs) showed no toxicity against the A549 cell line and demonstrated great potential for removing lead (Pb) from the environment. Similarly, Sikdar et al. conducted biosynthesis of IONPs using fruit residues with the aim of exploring their viability for industrial applications [4]. Segura et al. [5] used saponin extract from *Chenopodium quinoa* to obtain silver (Ag) NPs. It was demonstrated that saponin extract contains chemical groups that favored the reduction of Ag NPs. These examples highlight the interest in using biological resources to produce IONPs, which could have important implications in terms of both production efficiency and practical applications in various fields.

Within this context, quinoa (*Chenopodium quinoa*) has attracted particular attention due to its exceptionally rich composition of a variety of bioactive compounds, but it has not been used in the IONPs’ synthesis till now. These compounds include polyphenols, flavonoids, and phenolic compounds, which have been shown to have reducing and stabilizing properties in the IONPs’ synthesis [6,7,8,9,10,11]. This unique combination of active ingredients makes quinoa an ideal candidate to play a key role in IONPs’ synthesis. The inherent ability of quinoa to reduce and stabilize metal species during NPs’ synthesis not only offers an alternative, but a more sustainable route for nanoscale production of these materials. This can have applications in various fields ranging from biomedicine to material science and engineering. This connection between the biochemical richness of quinoa and its potential use for NPs’ synthesis underlines the importance of exploring and understanding natural resources more thoroughly in search of innovative and sustainable solutions in nanotechnology. It is worth mentioning that the preparation route of the quinoa extract (QE) will ensure the retention of their chemical groups, and therefore their components will act as reducers and stabilizers.

However, reducing agents present in the QE are composed of a complex matrix of components, such as amino acids, proteins, enzymes, vitamins, polyphenols, flavonoids, and polysaccharides [1,12]. Identifying the specific interactions between the QE components in the formation of IONPs is a challenging and complex task due to the diversity of biological compounds presented in the extract. For example, the commercial white quinoa extracts have nutritional value and cultural importance well-recognized, but they also have potential as a biological resource for the synthesis of IONPs is exploited. Therefore, this choice would represent a bridge between the traditional knowledge of the Andean communities and the cutting edge of scientific research, opening new possibilities for the application of quinoa in the synthesis of novel nanoarchitectures.

Consequently, in this research and for the first time, commercial white *Chenopodium quinoa*, a pseudocereal recognized for its excellent quality and nutritional value, especially grown in the Andean areas [13], was effectively chosen in the synthesis process of IONPs. Additionally, chemical precursors of iron sulfate (FeSO_4_) and iron chloride (FeCl_3_) were used in the biosynthesis, assisted by chemical co-precipitation of NPs. With the aim of determining the effectiveness of QE as an IONPs’ stabilizer and to establish the optimal amount of ammonium hydroxide (NH_4_OH) required to obtain the precipitate, ten (10) samples were systematically synthesized by modifying the QE concentration (% *w*/*v*). Biosynthesized NPs were characterized using a variety of physicochemical techniques that allowed to understand in details the structural, morphological, colloidal, vibrational, magnetic, and hyperfine properties of the resulting IONPs. The findings demonstrate the effectiveness of QE concentration in the size-controlled properties of NPs. A qualitative study for the mechanism of synthesis and functionalization using the QE was performed using infrared spectroscopy. The presented results contribute significantly to the advancement of knowledge in the field of nanotechnology and could have significant implications in a variety of fields, including medicine, industry, and the environment.

## 2. Materials and Methods

### 2.1. Procedure to Obtain Quinoa Extract

**Scheme 1:**
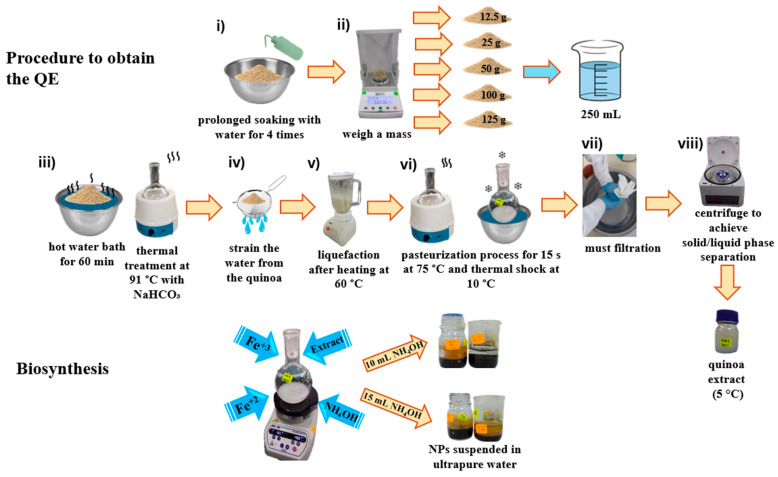
Pictorial representation of the QE preparation steps and biosynthesis.

The procedure has eight steps: (i) It begins with the selection of high-quality quinoa, preferably free of impurities. Quinoa is subjected to prolonged soaking for 4 h in a ratio of 1:2 (quinoa/water) to reduce the content of saponins, compounds that can impart an unwanted bitter taste, as observed by testing the flavor at laboratory conditions. This step is crucial to improving the organoleptic quality of the final extract (rich in proteins and active functional chemical groups). After soaking, quinoa is meticulously washed 4 to 5 times to remove any residue and foam textures, aiding in further removal of saponins and other impurities. (ii) Then, selected amounts (in grams) of quinoa (12.5 to 125 g) are weighed. (iii) Subsequently, a thermal treatment in a water bath for 60 min is carried out to remove excess water and prepare the quinoa for the next step. To reduce remnant bitter flavors and the residual content of saponins, a treatment with sodium bicarbonate (NaHCO₃) at 0.02% *w*/*v,* at 91 °C, and a mass of 40 mg is performed. (iv) Once the previous treatments are completed, quinoa is separated from the de-bittered solution and rinsed with cold water to halt cooking and preserve its characteristic texture and flavor. (v) Next, the liquefaction stage is carried out, where quinoa is blended with water in different quinoa mass and volume of water % *w*/*v* to obtain homogeneous extracts; see Equation (1).

(1) $$\left[\;Extract\;concentration\right]\%=\frac{Quinoa\;Mass}{\;{Vol.}_{H_{2}O}}\times 100\%$$

where ${Vol.}_{H_{2}O}$ is the water volume (250 mL) fixed during the extract preparation, as shown in Scheme 1.

Liquefaction is performed for 3 min at a vigorous speed, ensuring uniform dispersion of soluble components of quinoa in water. This stage is crucial to maximize the yield of the extract and to ensure uniform distribution of its nutritional and bioactive components. (vi) Then, the High Temperature Short Time Pasteurization (HTST) process is followed according to the International Dairy Association 2017 [14,15]; it has two sub-steps: (vi.1) the QE is heated at 75 °C for 15 s to inactivate any present pathogenic microorganism and (vi.2) a thermal shock is applied by immersing the container in water at 10 °C to halt any residual enzymatic activity and preserve the QE quality. This process is fundamental to ensuring the microbiological safety of the QE and extending its shelf life [16]. (vii) The obtained blend must is filtered, and (viii) the final QE solution is centrifuged at 4000 rpm for 5 min and stored in transparent glass containers inside a refrigerator at 5 °C and in dark conditions.

### 2.2. Biosynthesis of QEx Series

The biosynthesis of IONPs involved mixing FeSO_4_•7H_2_O (Merck KGaA, Darmstadt, Germany, purity > 99.5%) and FeCl_3_ (Sigma Aldrich, Burlington, MA, USA, 97% pure) in a molar ratio of 1:2 with addition of NH_4_OH at 28% *v*/*v* in 100 mL of QE using a magnetic stirrer at 400 rpm and 80 °C for 30 min. First, 5.2 g of FeSO_4_•7H_2_O was added, resulting in a dark green coloration, presumably caused by the contact of ferrous salt with the organic phase of the QE. Then, 6 g of FeCl_3_ was added, and the typical dark coloration corresponding to the formation of IONPs was observed. The NPs’ biosynthesis was performed by changing the amount (10 to 15 mL) of NH_4_OH under different QE concentrations (ten samples plus a control one), according to Table 1. Subsequently, the mixture was cooled down to 300 K and washed with ultrapure water until the pH stabilized at approximately 6.5–7. Finally, the solid was suspended in 50 mL of ultrapure water. The samples were dried at 70 °C in an oven for subsequent characterizations. They were labeled as QEx (x = 1–10) series.

### 2.3. Structural Properties: X-ray Diffraction Measurements

The X-ray diffraction (XRD) measurements were performed using an Empyrean diffractometer (Malvern Panalytical, Malvern, UK) operating with a Cu tube at setup conditions of 45 kV and 40 mA. The X-ray data were obtained using Bragg–Brentano geometry; CuK_α_ (λ = 1.54056 Å) in a range of 2*θ* = 10–80°. Phase identification was carried out using the software Match v3 [17]. The identified CIF files were #9006316 for maghemite and #9002158 for goethite. The FullProf Suite software (Gif sur Yvette Cedex, France, version January 2021) was used for the Rietveld refinement, where the Thompson–Cox–Hastings (TCH) pseudo-Voigt axial divergence asymmetry function was used as a function of the diffraction profile. Finally, the instrumental resolution function (IRF) of the diffractometer was obtained from the aluminum oxide standard (Al_2_O_3_) with the following parameters: U = 0.0093, V = −0.0051, and W = 0.0013 [18].

### 2.4. Morphological Properties: Transmission Electron Microscopy (TEM)

The analysis of particle size, size distribution, and morphology was conducted by a JEOL 2100F transmission electron microscope working at 200 kV in both transmission and high-resolution modes. The microscope was manufactured by JEOL in Tokyo, Japan. The particle size distribution (PSD) was determined by counting a total of 800 to 1000 particles from 30–35 images using the Image J software v 154. The histograms were fitted using a log-normal distribution [19]. Polydispersity values were determined by calculating the standard deviation of the log-normal distribution.

### 2.5. Dynamic Light Scattering and Zeta Potential Measurements

The powder samples weighing 30 mg were measured and then mixed with 50 mL of distilled water. The dispersion was exposed to sonication for a duration of 10 min at 300 K. Afterwards, the dispersion was filtered using a syringe filter with a porosity of 0.2 µm. It was then left resting for 5 min before being transferred into a plastic cell. The cell was then placed in the Nanobrook 90 Plus PALS equipment from Brookhaven Instruments (Nashua, NH, USA), which has the purpose of measuring dynamic light scattering (DLS) at a pH value of 7.

To conduct zeta potential measurements, a plastic container was filled with 20 mL of the dispersed sample. The pH of the sample was adjusted using titration equipment connected to the Brookhaven Nanobrook 90 Plus PALS equipment, ranging from 2 to 12.

### 2.6. Fourier Transmission Infrared Spectrometry (FTIR)

Transmission mode Fourier Transform Infrared Spectroscopy (FTIR) analysis was conducted on the samples using a Thermo Scientific Nicolet iS50 instrument (Waltham, MA, USA). The instrument had a resolution of 4 cm^−1^, and the analyses were performed within the wavenumber ranging from 4000 to 400 cm^−1^.

### 2.7. Magnetic Properties: Vibrating Sample Magnetometer (VSM)

Magnetic properties were measured with the Evercool II-Quantum Design Inc. (San Diego, California, USA). Physical Property Measurement System (PPMS) using the Vibrating Sample Magnetometry (VSM) module to obtain the magnetization curves, *M*(*H*), at 300 K and 5 K for a maximum scan field of 50 kOe. The law of approach to saturation (LAS) was employed on *M*(*H*) curves in the range of 20 to 50 kOe [19]. $M=M_{s}\left(1-\frac{b}{H^{2}}\right)+\chi H$_eff,_ where *M*_s_ is the saturation magnetization and *χ* is the high-field susceptibility; parameter *b* is related to the effective anisotropy coefficient (*K*_eff_) through $b=\frac{4}{15}{\left(\frac{K_{eff}}{M_{s}}\right)}^{2}$ [20].

## 3. Results and Discussions

### 3.1. XRD Results and Rietveld Analysis

**Figure 1:**
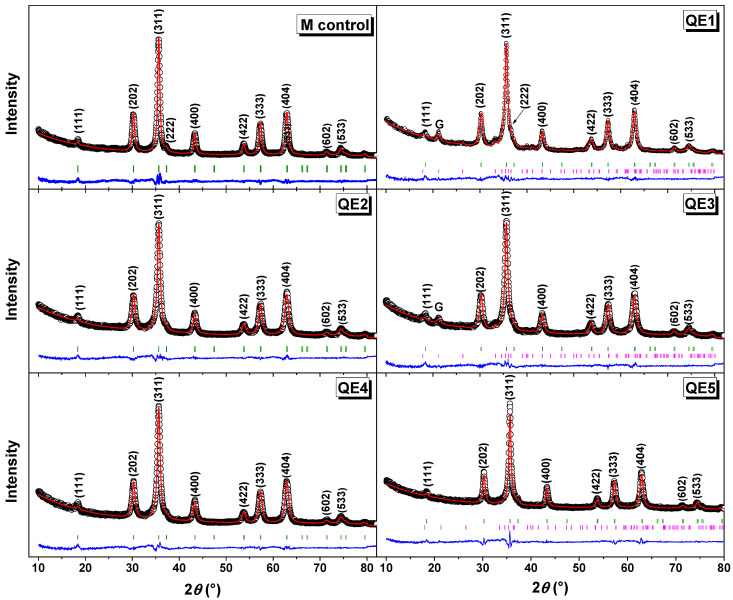
Refined X-ray diffractograms for the M control and QE (x = 1–5) samples. The black dots indicate the experimental data, the red lines are the refined model, and the blue line is the residual line. The vertical lines (green, magenta) indicate the Bragg’s peak positions. G stands for goethite.

**Figure 2:**
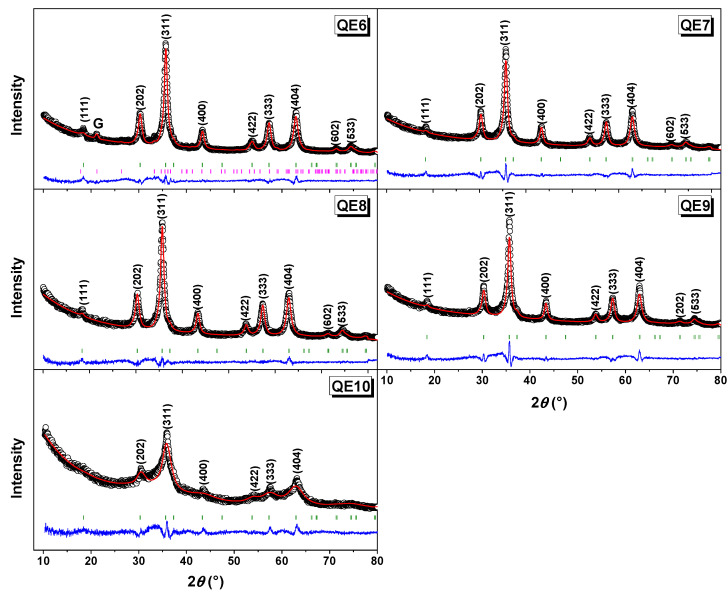
Refined X-ray diffractograms for the M control and QE (x = 6–10) samples.

Figure 1 and Figure 2 show the refined diffractograms using the Rietveld method. Table 2 summarizes the main crystallographic parameters obtained after the diffractogram refinements for the QEx series.

The crystallite size of the control sample showed a diameter of 8.9(1) nm and a single crystalline phase of γ-Fe_2_O_3_ (a = b = c = 8.355(1) Å). At low and intermediate QE concentrations, the synthesis produces a secondary phase of goethite, whose percentage composition varies between 5 and 10 wt %. From the refinement, it was possible to estimate the crystallite size of the goethite for the (101) Miller plane (more resolute diffraction peak). It varies from 9.4(1) to 11.8(1) nm with increase of the QE content. This secondary phase (goethite) has less crystallinity than the predominant phase of γ-Fe_2_O_3_, as supported by observing the lattice parameter for the γ-Fe_2_O_3_, which is constant despite the increments in the QE concentration; see Table 2. Nevertheless, the lattice parameter for goethite varied depending on the QE concentration, an effect that could indicate either its amorphous-like behavior and/or due to their extremely small sizes. The volume (mL) of added NH_4_OH has no significant influence when comparing samples with low QE concentrations. However, at high QE concentrations, the formation of single-phase γ-Fe_2_O_3_ indicates that synthesis can be improved independently of using a lower concentration of NH_4_OH. In other words, QE facilitates a lower use of the precipitating agent, probably due to the number of phenols in the QE, which facilitates the reduction of γ-Fe_2_O_3_ in aqueous medium.

Overall, the samples synthesized with QE at low and intermediate concentrations showed a decrease in the crystallite diameters compared to the control sample (8.9(1) nm). However, the QE10 sample with the highest QE concentration showed a considerably smaller crystallite diameter of 3.6 nm. These set of results indicates that the QE concentration induces a particle size control effect, as will be discussed below.

### 3.2. TEM Analysis

The main goal of this study was to obtain a pure nanomaghemite as a function of the QE concentration (more organic loading and less use of ammonium hydroxide, which is identified as a pollutant). Thus, the first step was to identify and differentiate, by X-ray diffraction and Rietveld analysis (as a conventional technique), the crystallographic phases found in the samples. The complementary analysis by other techniques in the subsequent sections was then focused on the samples with better crystallized behavior and less amount of precipitating agent. Furthermore, the crystallite diameter and point of zero charge (p.z.c.) were close and equal between QE7 and QE8 samples. More importantly, according to the literature, goethite interferes with the adsorption sites available on nanomaghemite surface for water remediation processes, for example, in the Pb(II) uptake from synthetic waters (see previous reference [21]). Hence, our characterization methodology is also aligned with our environmental purposes, in which these samples will be applied in a future work.

**Figure 3:**
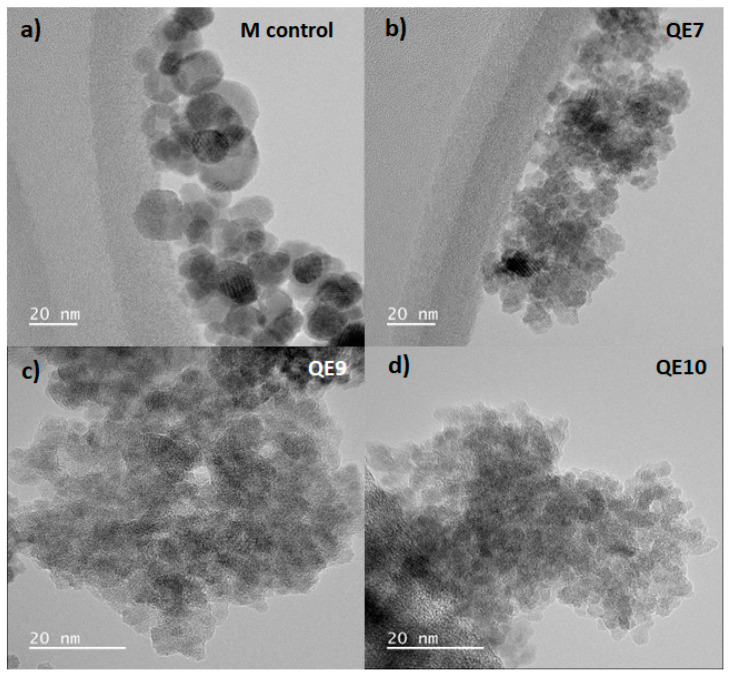
TEM images for the (**a**) M control, (**b**–**d**) QEx (x = 7, 9, and 10) samples.

For the above-mentioned reasons, the M control, QE7, QE9, and QE10 samples were chosen for the TEM analysis. Figure 3a–d shows the most representative images for the above-mentioned samples.

**Figure 4:**
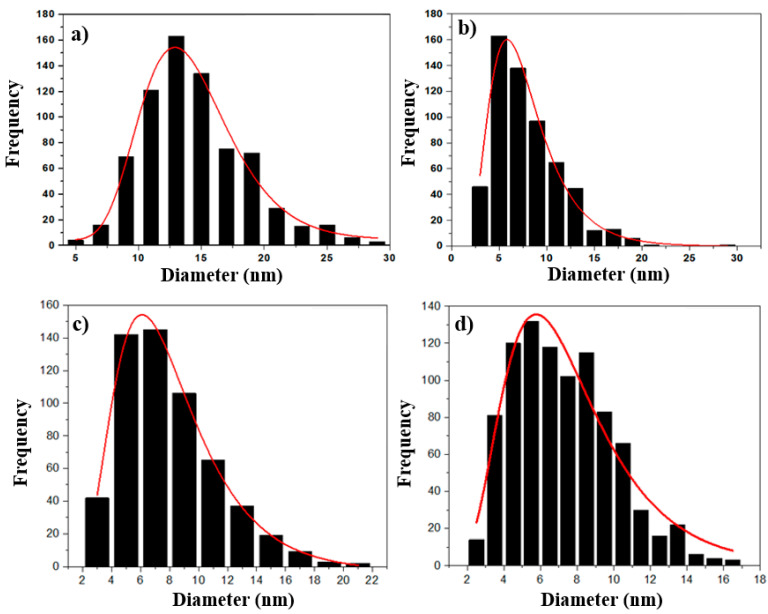
(**a**–**d**) PSD histograms of control and QEx (x = 7, 9, 10) samples. The red line indicates the log-normal distribution fit.

The sample synthesized, using the conventional method of chemical co-precipitation, produced NPs with a mean diameter of 13.8 nm; this was determined from the particle size distribution histogram. On the other hand, samples with the extract concentration of 40% *w*/*v* and samples QE8 and QE9 at 50% *w*/*v* showed diameters of less than 10 nm, as displayed in Table 3. Additionally, the polydisperse index (PDI) was calculated for these four samples, where the samples biosynthesized with the QE have a value of 0.06 in contrast to the value of 0.02 in the M control sample. This variability in the size of biosynthesized NPs is related to the presence of the organic composition of the QE that functionalized the particle surface. White quinoa is rich in phenolic chemical compounds, which act as reducing agents and particle size controllers. The asymmetry of log-normal distributions allowed to corroborate the asymmetric and typical distribution in NPs’ counts, which is clearly observed in Figure 4. It should be noted that it is very important to have good statistical counting to be able to model the trend of the distribution, and thus, to obtain the corresponding statistics; as seen in Table 3.

By comparing with other systems biosynthesized with various extracts (see Table 4), it can be concluded that the biogenic nature of organic compounds has an influence on the size distribution of NPs, which usually varies between 2.3 and 60 nm. However, the use of QE allows average diameters less than 10 nm and more important, we do not need to use a high amount of NH_4_OH as often occurs in ordinary co-precipitation processes used to get IONPs.

### 3.3. DLS and Zeta Potential Analysis

**Figure 5:**
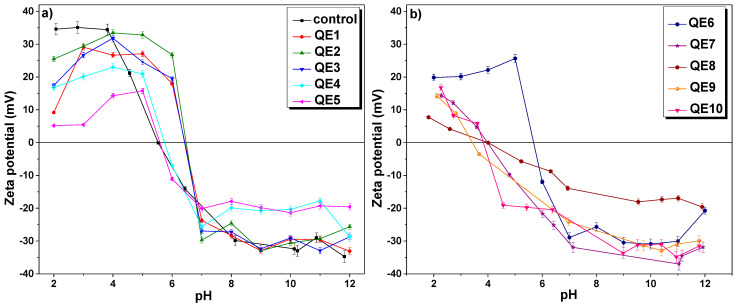
(**a**,**b**) Zeta potential measurements for QEx series.

The results of hydrodynamic diameter and zeta potential measurements are shown in Figure 5a,b and Table 5.

Table 5 shows the concentrations of the prepared suspensions for the measurement of the colloidal properties, as well as the values for the hydrodynamic diameter and the point of zero charge (p.z.c.) determined in Figure 5a,b. The *D*_H_ value of the M control sample differs from the other values of the samples with QE. Indeed, the QE samples have a varied *D*_H_ value related to the polydisperse nature of the suspended dust. It is important to mention that magnetic nanoparticles, or IONPs, agglomerate due to the presence of magnetic dipolar interaction, which competes with Van der Walls forces in aquatic media [22]. The Lewis acid-type behavior of the IONPs indicates that IONPs may present colloidal stability in an aqueous environment; depending on the pH and ionic strength, i.e., the IONPs may be charged with positive or negative electrostatic potential [23]. The value of the pH that separates this characteristic behavior is defined as the isoelectric point or point of zero charge.

The value of the p.z.c. for the M control sample and for the samples biosynthesized with the QE occurs at an acid pH value < 7. Samples with higher QE concentration values at 40 and 50% of *w*/*v* indicate a value of p.z.c. decreasing to an acidic pH. This result may be related to the decrease in particle diameter relative to the M control sample.

### 3.4. FTIR and Thermogravimetric Analysis

The functional groups of powder quinoa and IONPs were identified using their infrared spectra. For this purpose, the region between 4000 and 400 cm^−1^ was analyzed. First, the reference [24] was used to study the effect of relative humidity on the optical properties of *Chenopodium quinoa* Willd flour. In our case, the band of the water molecule was found at the position 3294 cm^−1^, characteristic of the symmetrical molecular vibration of the hydroxyl groups (OH), corresponding to the white powder quinoa. On the other hand, the species *Chenopodium quinoa*, in its form of feces and flour, shows characteristic IR bands, according to Table 6.

**Figure 6:**
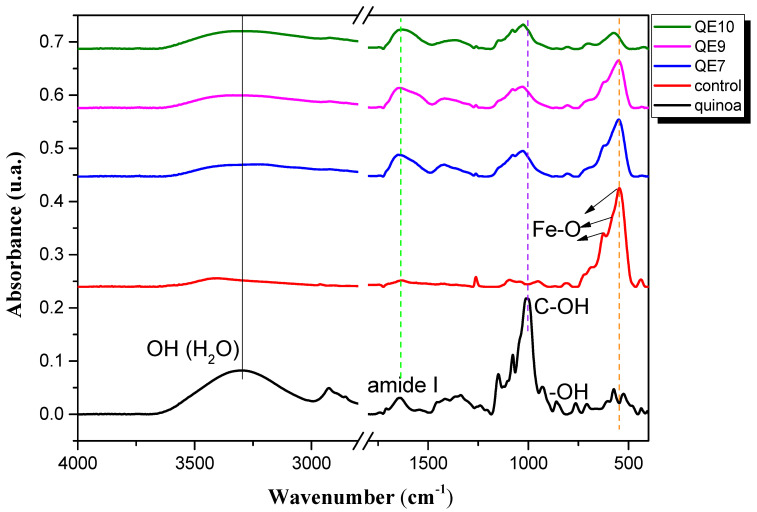
FTIR spectra for the M control, pure quinoa, and QEx (x = 7, 9, and 10) samples.

Figure 6 shows the FTIR spectra of the analyzed samples and their corresponding wave number positions. The M control sample displayed IR bands characteristic of tetrahedral and octahedral sites in the region from 430 to 640 cm^−1^ [26,27]. The most intense peak is related to the tetrahedral site at 547 cm^−1^ and a lower-intensity shoulder at 580 cm^−1^. On the other hand, the peak located in the wave number of 626 cm^−1^ is related to the octahedral site. This supports the results obtained by XRD, where the single phase of maghemite was observed in the M control sample.

When analyzing the biosynthesized samples with QE, the vibrational bands characteristic of maghemite and white quinoa were observed concomitantly. The most outstanding wave number positions are shown in Table 7.

The most intense IR band for powdered quinoa was observed at 1001 cm^−1^. However, for samples with higher concentrations of QE, they showed a shift towards 1026 cm^−1^, suggesting a chemical interaction between the functional groups of Fe-O and the -COOH polymer groups (functionalization of particle surface). The amide I and C–N amino acid groups also showed significant corrections in their IR positions. Therefore, a linking mechanism could be proposed between the various compounds of white quinoa and the surface of Fe-oxide.

**Figure 7:**
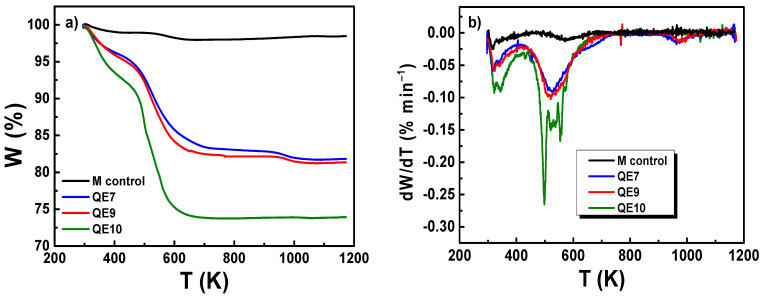
(**a**) Weight loss (W) vs. temperature and (**b**) their derivative weight loss (dW/dT) curves for M control and QEx (x = 7, 9, and 10) samples.

TGA results (Figure 7a) show that an increase in QE concentration leads to a higher organic loading, which in turn enriches the presence of flavonoids during biosynthesis. The total weight loss increases from 2% (M control) to 19% (QE7, QE9) and 27% (QE10). Figure 7b displays three distinct peaks for nearly all the samples, with the second signal of the QE10 sample exhibiting a noisy response. The first peak corresponds to the physiosorbed water that the sample retained during synthesis, while the second broad peak indicates the organic loading on the IONPs surface, indicating the flavonoids and proteins associated with QE. More importantly, the peak area increases as QE concentrations increase, indicating that the QE10 sample has the largest fraction of polyphenols and organic matter. Finally, the small peak around 950 K was likely linked to the change from γ-Fe_2_O_3_ to α-Fe_2_O_3_ (this peak is hidden in the M control sample because of the noisy signal) [28]. This shows that, the γ-Fe_2_O_3_ to α-Fe_2_O_3_ transition can move to higher temperatures when the organic loading goes up, with 1200 K being the highest temperature our setup could handle.

### 3.5. Magnetic Analysis

**Figure 8:**
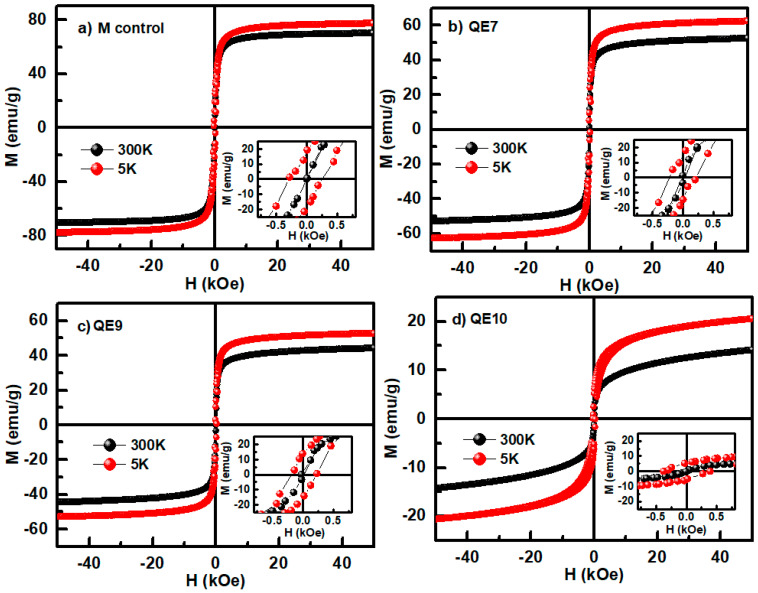
*M*(*H*) curves for (**a**) M control, (**b**) QE7, (**c**) QE9, and (**d**) QE10 samples. The inset displays the zoomed region for the magnetic field amplitude of ±0.5 kOe.

Figure 8a–d shows the *M*(*H*) curves at 300 and 5 K for the M control and QEx (x = 7, 9, and 10) samples. The characteristic S-shape for ferrimagnetic particles is observed. The *M*(*H*) curves for the M control and QEx (x = 7 and 9) samples display a saturated behavior, indicating blocked particles with bigger crystallized sizes in agreement with the Rietveld refinement. The QE10 sample, on the other hand, exhibits a non-saturated behavior, which is expected for particles smaller than 8 nm [27]. As a result, the biosynthesis has a significant influence on the NPs’ magnetic response.

Table 8 summarizes the magnetic parameters obtained by the LAS fit. When increasing the organic loading, the *M*_s_ values decreased from 69 to 11 emu g^−1^ (QE10), which corresponds to 84% of the expected value of a crystallized sample (bulk-like sample), indicating a large non-magnetic contribution as expected for an organic functionalization of particle surface and particle size reduction observed by TGA and TEM data. Also, it appears that the NH_4_OH content significantly influences the magnetic seed crystallization and PSD, since this last sample was observed to have a broad PSD (close to a multimodal distribution, see Figure 4d) in comparison to the M control and QEx (x = 7 and 9) samples. These observations above-described reflect the difference in *M*_s_ values between QE9 and QE10 samples. The value of *K*_eff_ was also influenced by the size reduction in the magnetic core observed by TEM, which was one order higher than a bulk magnetic sample (4.7 × 10^3^ J m^−3^) [29,30].

### 3.6. Functionalization Mechanism

Pereira et al. [31] used chromatographic evaluation to study white commercial quinoa. It reveals a complex molecular structure where two main flavonoid molecules were observed. These are quercetin and kaempferol glycoside derivatives; their intensity profiles and concentrations depend on their variety, cultivars, and accessions. While both quercetin and kaempferol have a 3-hydroxy flavone backbone, quercetin differs from kaempferol in that, because it has an extra hydroxyl group (–OH) at the R1 position [32].

The active IR bands of quercetin are mainly related to carboxyl(–COOH) and carbonyl(C=O) groups in the phenyl skeleton located at ca. 1015 (medium intensity) and 1664 (strong) cm^−1^ [33], while kaempferol exhibits these IR bands in a widely IR region at 1016–1029 cm^−1^ (–COOH) and 1632–1647 cm^−1^ (C=O), respectively [34]. Our QEx (x = 7, 9, and 10) samples only show the presence of the –COOH groups with the absence of the C=O group, hence indicating that the chemical interaction is favored by the carboxyl groups.

The –OH groups have been observed to functionalize the IONPs using different polymers such as polyethylene glycol, dextran, polyvinyl alcohol, and pluronic [35]. However, the use of quinoa extracts to coat the IONPs’ surface with hydroxyl groups has not been reported. Chemically speaking, kaempferol is a more stable molecule and is less reactive. Hence, it can be used to explain the biosynthesis mechanism and surface hydroxyl functionalization occurring at the IONPs’ surface. Magnetic NPs behave as a Lewis base depending on the p.z.c.; above this value, deprotonation occurs according to the following chemical reaction (see Equation (2)) [36]:

(2) $$\equiv Fe-OH⇌\equiv Fe-O^{-}+H^{+}$$

**Scheme 2:**
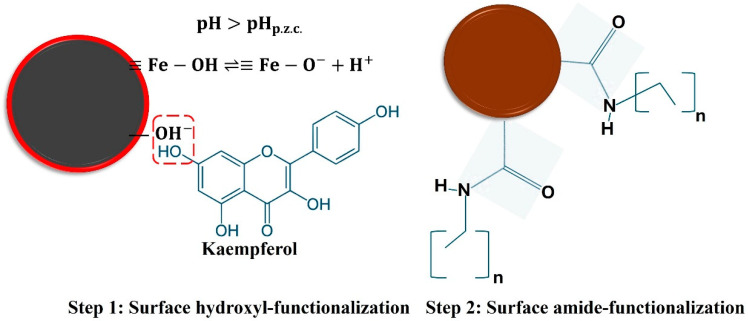
Functionalization mechanism for the QEx series.

The p.z.c. values in Table 5 indicate that alkaline mediums favor hydroxyl coordination. As the QE concentration increases, the p.z.c. shifts to the left of the X-axis, reaching 3.8 for 50% *w*/*v*. This observation suggests that high QE concentrations promote surface hydroxyl functionalization. Scheme 2 shows the first step in the biosynthesis mechanism governed by the hydroxyl groups presented in kaempferol.

On the other hand, amide I and amide III chemical groups found in the QE proteins also functionalize the IONPs surface through the carbonyl groups found in amide I chemical groups; see Table 7 and reference [37]. Scheme 2 shows the possible mechanism of functionalization occurring during NPs’ biosynthesis.

## 4. Conclusions

Surface hydroxyl- and amide-functionalized maghemite nanoparticles were synthesized using diverse concentrations of commercial white QE. The QE was prepared by a purification method involving eight steps highlighting the HTST process, filtration, centrifugation, and storage at laboratory conditions. The refined diffractograms and statistical parameters revealed that the crystallite sizes were significantly smaller for the highest concentration of 50% *w*/*v* and the lowest volume of NH_4_OH (10 mL). Furthermore, all the samples had the same refined lattice parameter for γ-Fe_2_O_3_ (a = b = c = 8.355(1) Å), showing that they were highly crystalline and stoichiometric samples that preserved the inverse spinel cubic structure. However, we found a dependence between the QE concentration and the percentage phase of the crystallographic phases. A low fraction of a secondary phase of goethite (5 and 10 wt%) was observed at low and intermediate QE concentrations. Increasing the QE concentration, this issue was definitely resolved when the amount of the precipitating agent was reduced, which led to an improvement of the synthesis process (less contaminants are available at the process end). The biosynthesized IONPs (at high QE concentrations) had sizes ranging from 7 to 8 nm, compared to the M control synthesized by conventional coprecipitation. Therefore, this is a great contribution of this work, as it uses a commercial Peruvian product that can facilitate the development of a magnetic nanomaterial with very small dimensions and high crystallinity (often a problem with amorphous-like samples obtained by biosynthesis), without considering that the material adsorbs the functional groups of the QE and could facilitate its functionalization with other compounds for various technological applications. The TGA results supported the organic loading with increasing QE concentration. Increasing the organic loading shifted the thermal $\gamma \;to\;\alpha {-Fe}_{2}O_{3}$ transition to higher temperatures. The p.z.c. values also showed a reduction from 5.5 for the M control to 3.4 (or 3.8) for the IONPs when using 50% *w*/*v*, suggesting the presence of hydroxyl groups on the IONPs’ surface. Biosynthesis also affected the magnetic behavior, with the QE10 sample exhibiting the smallest *M*_s_ value of 11 emu g^−1^. The hard-like magnetic behavior was associated with an increase in *K*_eff_, which was found to be 1.3 × 10^4^ J m^−3^. Finally, FTIR measurements confirmed the presence of -COOH, amide I, and amide III chemical groups, indicating that the IONPs’ surface was modified and stabilized with zeta potential values above the threshold stability (>−30 mV). Hence, the use of QE during the IONPs’ biosynthesis not only reduces the use of hazardous chemicals, but also represents a green and environmentally friendly approach to obtain one-phase crystallized IONPs.

## Figures and Tables

**Table 1:** Chemical compounds and their quantities used during biosynthesis.

% *w*/*v*	Sample	QE (mL)	H_2_O	FeSO_4_•7H_2_O (g)	FeCl_3_ (g)	NH_4_OH (mL)
0	M control	0	100 mL	5.2	6	20
5	QE1	100	0	5.2	6	10
5	QE2	100	0	5.2	6	15
10	QE3	100	0	5.2	6	10
10	QE4	100	0	5.2	6	15
20	QE5	100	0	5.2	6	10
20	QE6	100	0	5.2	6	15
40	QE7	100	0	5.2	6	10
40	QE8	100	0	5.2	6	15
50	QE9	100	0	5.2	6	10
50	QE10	100	0	5.2	6	15

**Table 2:** Rietveld refinement parameters, statistical parameters, and crystallite sizes of the M control and QEx (x = 1–10) samples. R_p_ (%), R_wp_ (%), and R_exp_ (%) are the profile residual, the weighted profile residual, and the expected profile residual, respectively, while χ^2^ is the goodness of the refinement. M indicates maghemite and G goethite.

Sample	Phase	Caglioti Parameters			Lattice Parameters (Å)	*D*_XRD_ (nm)	Weight Fraction (%)	Statistical Parameters
		U	V	W				
M control	γ-Fe_2_O_3_	0.013	−0.034	0.008	a = 8.355(1) b = 8.355(1) c = 8.355(1) α = β = γ = 90°	8.9(1)	100.0	R_p_ = 14.3% R_wp_ = 14.0% R_exp_ = 7.2% χ^2^ = 3.8
QE1	γ-Fe_2_O_3_	0.013	−0.034	0.008	a = 8.355(1) b = 8.355(1) c = 8.355(1) α = β = γ = 90°	7.2(1)	90.0	R_p_ = 13.5% R_wp_ = 13.6% R_exp_ = 8.3% χ^2^ = 2.7
	G	0.113	−0.584	0.561	a = 9.952(1) b = 3.026(1) c = 4.616(1) α = β = γ = 90°	10.4(1).	10.0	
QE2	γ-Fe_2_O_3_	0.013	−0.034	0.008	a = 8.355(1) b = 8.355(1) c = 8.355(1) α = β = γ = 90°	7.5(1)	100.0	R_p_ = 13.2% R_wp_ = 13.3% R_exp_ = 8.1% χ^2^ = 2.7
QE3	γ-Fe_2_O_3_	0.013	−0.034	0.008	a = 8.355(1) b = 8.355(1) c = 8.355(1) α = β = γ = 90°	7.5(1)	93.0	R_p_ = 13.3% R_wp_ = 13.3% R_exp_ = 8.0% χ^2^ = 2.8
	G	0.702	−0.670	0.626	a = 9.945(1) b = 3.035(1) c = 4.606(1) α = β = γ = 90°	9.4(1)	7.0	
QE4	γ-Fe_2_O_3_	0.013	−0.034	0.008	a = 8.355(1) b = 8.355(1) c = 8.355(1) α = β = γ = 90°	7.1(1)	100.0	R_p_ = 13.1% R_wp_ = 13.0% R_exp_ = 7.7% χ^2^ = 2.9
QE5	γ-Fe_2_O_3_	0.013	−0.034	0.008	a = 8.355(1) b = 8.355(1) c = 8.355(1) α = β = γ = 90°	9.4(1)	95.0	R_p_ = 19.9% R_wp_ = 17.3% R_exp_ = 9.1% χ^2^ = 3.6
	G	0.436	−0.729	0.669	a = 9.892(1) b = 3.049(1) c = 4.587(1) α = β = γ = 90°	10.9(1)	5.0	
QE6	γ-Fe_2_O_3_	0.013	−0.034	0.008	a = 8.355(1) b = 8.355(1) c = 8.355(1) α = β = γ = 90°	6.5(1)	95.0	R_p_ = 16.5% R_wp_= 15.5% R_exp_ = 8.2% χ^2^ = 3.6
	G	0.113	−0.584	0.561	a = 9.952(1) b = 3.026(1) c = 4.616(1) α = β = γ = 90°	11.8(1)	5.0	
QE7	γ-Fe_2_O_3_	0.013	−0.034	0.008	a = 8.355(1) b = 8.355(1) c = 8.355(1) α = β = γ = 90°	6.3(1)	100.0	R_p_ = 21.6% R_wp_ = 19.4% R_exp_ = 9.9% χ^2^ = 3.9
QE8	γ-Fe_2_O_3_	0.013	−0.034	0.008	a = 8.355(1) b = 8.355(1) c = 8.355(1) α = β = γ = 90	6.9(1)	100.0	R_p_ = 15.4% R_wp_ = 14.6% R_exp_ = 10.0% χ^2^ = 2.1
QE9	γ-Fe_2_O_3_	0.009	−0.005	0.001	a = 8.355(1) b = 8.355(1) c = 8.355(1) α = β = γ = 90	7.6(1)	100.0	R_p_ = 26.3% R_wp_ = 23.3% R_exp_ = 10.2% χ^2^ = 5.2
QE10	γ-Fe_2_O_3_	0.009	−0.005	0.001	a = 8.354(1) b = 8.354(1) c = 8.354(1) α = β = γ = 90	3.6(1)	100.0	R_p_ = 15.7% R_wp_ = 15.3% R_exp_ = 9.1% χ^2^ = 2.8

**Table 3:** Morphological parameters obtained from the fit of the PSD histograms. *D* is particle diameter (nm), *σ* is the standard deviation, PDI is the polydisperse index, and *n* is the number of particles.

Sample	*D*	*σ*	PDI	*n*
Control	13.8(2)	0.27	0.02	726
QE7	7.2(2)	0.45	0.06	587
QE9	7.5(4)	0.45	0.06	570
QE10	7.0(2)	0.40	0.06	914

**Table 4:** Comparison between particle type, extract used, and particle diameter for various nanosystems reported in the literature.

Nanosystem	Extract	Particle Diameter (nm)	Reference
Superparamagnetic iron-oxide nanoparticles	*Eucalyptus globulus*	2.3	[6]
nanomagnetite (Fe_3_O_4_)	*Rhus Coriaria*	7.9	[7]
Fe_3_O_4_	*Artemisia leaves*	19–24	[8]
Iron-oxide NPs (Fe_3_O_4_)	*Clausena indica*	47.5–59.5	[9]
Iron-oxide NPs	*Carica papaya*	21.6	[10]
Magnetic nanospheres of α-Fe_2_O_3_	*Bridelia retusa*	38.6	[11]

**Table 5:** Colloidal parameters for the zeta potential measurements of QEx samples. The concentrations of the prepared suspensions were varied to obtain the best physical conditions for DLS measurements, i.e., to reproduce the Brownian movement among suspended particles and avoid its flocculation and a bias determination of their colloidal properties.

Sample	Concentration (g L^−1^)	*D*_H_ (nm)	p.z.c.
M control	1.0	136	5.5
QE1	7.5	242	6.4
QE2	5.8	263	6.5
QE3	7.3	349	6.4
QE4	7.4	1310	5.8
QE5	3.6	417	5.6
QE6	3.5	1101	5.7
QE7	6.4	305	4.0
QE8	4.5	649	4.0
QE9	0.5	396	3.4
QE10	0.5	383	3.8

**Table 6:** Positions of wave numbers in the IR region for quinoa in Ref. [25] and in the white powder quinoa.

Functional Groups	Wavenumber (cm^−1^) (Literature)	Wavenumber (cm^−1^) (This Work)	Band Intensity
–CH	2915	2923(8)	Weak
–CH_2_	2850	2850(0)	Weak
–COOH	998	1001(3)	Strong (characteristic)
O–H	855	860(5)	medium
C=O	1737	-	weak
Amide I	1640	1639(1)	medium
Amide II	1545	-	weak
C–N	1237	1240(3)	weak

**Table 7:** IR bands and wavenumber positions (cm^−1^) for the QE7, QE9, and QE10 samples.

QE7	QE9	QE10	IR Band
545	545	565	γ-Fe_2_O_3_ (strong)
624	624	694	γ-Fe_2_O_3_ (strong)
1026	1026	1026	Quinoa (-COOH) (strong)
1647	1637	1628	Quinoa (amide I) (strong)
1261	1263	1263	C–N (amide III)

**Table 8:** Magnetic parameters obtained for the M control and QEx samples fitting the *M*(*H*) curves with LAS approach.

Sample	T (K)	*H*_c_ (kOe)	*M*_s_ (emu g^−1^)	*K*_eff_ (10^4^ J m^−3^)
M control	5	0.27(1)	76	4.0
	300	-	69	3.2
QE7	5	0.22(1)	64	3.3
	300	-	51	2.8
QE9	5	0.21(1)	51	2.9
	300	-	42	2.6
QE10	5	0.36(1)	17	1.8
	300	-	11	1.3

## Data Availability

The original data related to this research can be requested at any time by sending an email to the corresponding author: juan.ramos5@unmsm.edu.pe.
